# Coping Strategies for Adverse Effects of Antiretroviral Therapy among Adult HIV Patients Attending University of Gondar Referral Hospital, Gondar, Northwest Ethiopia: A Cross-Sectional Study

**DOI:** 10.1155/2018/1879198

**Published:** 2018-12-02

**Authors:** Yitayih Kefale Gelaw, Boressa Adugna, Adino Tesfahun Tsegaye, Tadesse Melaku, Belayneh Kefale

**Affiliations:** ^1^Department of Clinical Pharmacy, Enat Hospital, Amhara Region, North Shewa, Alem Ketema, Ethiopia; ^2^Department of Clinical Pharmacy, School of Pharmacy, College of Medicine and Health Sciences, University of Gondar, Gondar, Ethiopia; ^3^Department of Epidemiology and Biostatistics, School of Public Health, College of Medicine and Health Sciences, University of Gondar, Gondar, Ethiopia; ^4^Department of Pharmacy, College of Medicine and Health Science, Ambo University, P.O. Box 19, Ambo, Ethiopia

## Abstract

**Background:**

Adverse effects from antiretroviral therapy (ART) have an impact on quality of life and medication adherence. There is no clear understanding of how people manage the adverse effects of ART. The individual taking medications which cause serious adverse effects may choose to stop or reduce the medications to relieve the adverse effects. Hence, this study was aimed at assessing coping strategies for adverse effects of ART among adult human immunodeficiency virus (HIV) patients.

**Methods:**

A cross-sectional study was conducted at HIV clinic of University of Gondar Referral Hospital (UoGRH). A total of 394 study participants were recruited by systematic random sampling. Data were collected through interviewing patients. Data were entered to Epi-Info 3.5.4 and analyzed using SPSS-20.0. Descriptive statistics were used to summarize patient's sociodemographic data and the adverse effects of their ART regimen. Binary and multivariate logistic regressions were used to investigate the potential predictors of nonadherence coping strategies.

**Results:**

The majorities of study participants were females (66%) and aged between 35 and 44 years (38.1%). The major adverse effects reported by the participants were headache (48.2%) followed by fatigability (18%) and loss of appetite (17.5%). Coping strategies used by HIV patients for adverse effect of ART were positive emotion coping strategy (91.1%), social support seeking (76.6%), taking other medications (76.6%), information seeking (48.7%), and nonadherence (35.5%). Younger age (AOR = 29.54, 95% CI = 2.49–35.25, p = 0.007), low level of education (AOR = 5.70, 95% CI = 2.16-15.05, p < 0.001), and living far from the health institution (AOR = 2.68, 95% CI = 1.29–5.57, p = 0.008) were associated with nonadherence coping strategy to relieve the adverse effects of ART.

**Conclusion:**

The present study revealed that positive emotion coping was the most commonly used strategy. Age, level of education, and distance from health institution were the predictors of nonadherence coping strategy.

## 1. Introduction

Adverse drug reactions (ADRs) are any noxious, unintended, and undesired effect of a drug, which occurs at doses used in humans for prophylaxis, diagnosis, or therapy, or for the modification of physiological functions [1, 2], while symptoms are subjective complaint that is reported by patients due to some disease state or medications. Adverse effects from ART are common and were the most common reason for switching or discontinuing therapy and for medication nonadherence [3, 4]. Adverse effects are not typically life-threatening but can impact the quality of life (QoL), negatively affect patients' willingness to adhere to their regimens, and influence decisions about healthcare [5, 6]. A better understanding of how patients cope with undesirable adverse effects from treatment can inform interventions to remediate the negative impact of adverse effects on treatment adherence and QoL. Coping is defined as the cognitive and behavioral responses that an individual employs to deal with the stressors. Coping can take the form of active behavior (problem-focused), regulation of distress (emotion-focused), or the maintenance of well-being (meaning-based) [7]. One of the major challenges for patient being nonadherent to their ART is the incidence of ADRs. The main coping strategies for adverse effect of ART include nonadherence, social support seeking, using positive emotion, information seeking, and taking other medications [4, 7–12].

There was evidence that ADRs prevalence and incidence in ART programs are much higher and have a wide range of manifestations. Various studies demonstrate that adverse effect of ART regimens was the main reason for nonadherence [13, 14]. Nonadherence to ART regimens is common, leading to considerable deterioration of the disease and enhanced healthcare expenditure. Though nonadherence coping strategy in ART is an increasing problem for patients with HIV, it has not been extensively studied in patients with HIV. Previous studies have reported that 59.38% [15], 14.5% [3], and 17.3% [16] of HIV patients were nonadherent in United States, Nepal, and Ethiopia, respectively, and avoidance of adverse effect was the main reason contributing to nonadherence. Previous study in South Africa demonstrated that the primary reason for ART regimen changes was adverse effect related to drugs [17]. The prevalence and incidence of ADR in ART is rising globally [18]. Its incidence in different studies was found in 43.8% [19] and 6.3% [18] of HIV patients with ART in India and Nigeria, respectively. Studies in India reported ADR prevalence of 39.7% in intensively monitored ART patients [20]. In Ethiopia among ADR reports from 2002 to 2007, ADRs from ART contribute about 70.6% [6] and in other studies in Harar, Northern Ethiopia, and Gondar the prevalence of ADR with ART was 17%, 19%, and 89.8%, respectively [13, 21, 22]. Nonadherence will lead to higher viral load, drug resistance, and treatment failure. Evidence-based research that evaluates how often patients use different coping strategies to counteract the adverse effects of ART in developing countries such as Ethiopia is scanty [22]. Thus, there should be a continuing need to routinely assess coping strategies and their contributing factors for patients with HIV in health facilities [8, 23]. This is particularly imperative in resource-limited countries, with the predominance of economic instability and inadequate follow-up that might contribute to the incidence of poor medication adherence. Therefore, this study aimed to assess coping strategies for adverse effects of ART among adult HIV patients attending the UoGRH.

## 2. Methods

### 2.1. Study Design and Setting

A cross-sectional study design was conducted from February to March 2017 at UoGRH HIV clinic, Northwest Ethiopia. The hospital gives different inpatient and outpatient services for the community (around seven million catchment population). This hospital renders comprehensive HIV related services including voluntary counseling and testing (VCT), provider initiated testing and counseling (PITC), prevention of mother to child transmission (PMTCT), and ART program. Currently, there are about 5194 HIV patients actively attending ART in the hospital, having the daily patient flow of 150 on average.

### 2.2. Study Subjects

Adults with age ≥ 18 years who were on ART for at least one month and those who came for refill during the period of data collection and who had ART associated adverse effects were included in the study. ART associated adverse effects, which are a harmful or abnormal result caused by administration of medication (ART), were the most common reason for switching or discontinuing therapy and for medication nonadherence.

### 2.3. Sample Size and Sampling Methods

Sample size was computed by using single population proportion formula as follows:

(1) $$\begin{array}{c} n=\frac{Z_{{\left(\alpha /2\right)}^{2}}p\left(1-p\right)}{d^{2}} \end{array}$$

where *n* is expected sample size for population >10,000; *α*/2 is the critical value of a 95% confidence level interval (which corresponds to 1.96); and *P* means that we use positive prevalence estimated, to maximize sample size. Negative prevalence = 1 − 0.5 = 0.5, d = marginal error (d = 0.05); then the sample size is

(2) $$\begin{array}{c} n=\frac{1.96^{2}0.5\left(1-0.5\right)}{{\left(0.05\right)}^{2}}=384.16=\sim 384 \end{array}$$

Since the source of population was less than 10,000 (N=5194), reduction formula was employed to compute the final sample size. The corrected sample size, using the following correction formula, was 357.6~358,

(3) $$\begin{array}{c} \text{Corrected sample size}=\frac{n\times N}{n+N} \end{array}$$

Accordingly, the final sample size with 10% contingency was found to be 394.

A systematic random sampling method was employed to recruit the study participants in each day of the data collection process.

### 2.4. Data Collection Techniques

Adequate training was given for three pharmacists, who were recruited as data collectors. The training comprised uniform interpretation of the structured questionnaires, strict use of study criterion, explanation of the study objectives, getting oral consents, implementation of sampling technique, and maintaining confidentiality of the collected data. Data were collected by interviewing HIV patients who visited the HIV clinic during the study period using a validated structured questionnaire composed of closed and open ended questions. The structured questionnaire was assessed by an expert in the field of ART for clarity and comprehensiveness of its contents. The questionnaire was first prepared in English and translated to local language (Amharic) for ease of understanding. A pilot study was done with 5% (20) of the study participants and all the necessary modifications were done before implementing in the main study.

### 2.5. Data Analysis

Data were entered into Epi-Info and analyzed by using SPSS version 20.0 statistical software. Patients' baseline sociodemographic data and the frequency of major adverse effects were summarized using descriptive statistics. Univariable and multivariable binary logistic regression were conducted to determine the potential predictors of nonadherence coping strategy. Confidence interval (95%) and p value were used or reported in each logistic regression analysis. p value ≤ 0.05 was considered as statistically significant.

### 2.6. Ethical Consideration

Ethical clearance was obtained from Ethical Approval Committee, University of Gondar, College of Medicine and Health Sciences. Letter of permission was submitted to University of Gondar Referral Hospital medical director office prior to the beginning of the study. Verbal informed consent was obtained from participants before conducting the study. Participants were informed that their participation was voluntary and they can withdraw from the study at any stage if they desire without any penalty. Confidentiality of participants was maintained at all levels of the study and the name and address of the patient were omitted from the questionnaire.

## 3. Results

### 3.1. Sociodemographic Characteristics of HIV Patients

Three hundred and ninety-four HIV patients were involved in the study. About two-thirds of study participants were females and majorities (38.1%) of the study participants were within the age group of 35–44 years. Mean age of the study participants was 38.3 (SD = 9.8) years. Being married (51.8%) and orthodox (78.7%) participants accounted for the highest percentage. Being merchant (25.1%) and government employee (22.1%) accounted for the highest percentage of occupation. Education-wise, 25.4% attended primary and higher education (Table 1).

### 3.2. Clinical Characteristics and Health Conditions

The median CD4 count before ART initiation among the study participants, who faced ART adverse effect, was 153.5 with IQR of 624, while the most recent CD4 count was 384.5 with IQR of 408.8. The majority (43.9%) of the study participants were World Health Organization (WHO) clinical stage III at the time of starting ART, while at the time of data collection majority (95.7%) of study participants were treatment stage 1 (T1). Baseline functional status of the study participants was majorly working (77.9%) while a significant number of patients had ambulatory and bedridden functional status. Nonetheless, the current functional status for almost all (98.7%) of HIV patients was working (Table 2).

### 3.3. Reasons for Changing ART Regimen

Based on documented reasons from the patient's chart, 118 (29.9%) patients who started ART regimens were changed due to different reasons. Adverse effects (61.9%) were the most common reason for regimen change. Of 118 changed regimens, majority (64, 54.2%) of them were on D4T based ART regimen followed by AZT (34, 28.8%) based regimen and TDF (20, 17%) (Table 3).

### 3.4. Adverse Effects Experienced by HIV Patients

A total of 880 adverse effects were reported which was on average greater than two adverse effects experienced per patient and the majority of adverse effects were central nervous systems (CNS) and peripheral nervous systems (PNS) (62%), metabolic disorders (16.4%), gastrointestinal (GI) (8.6%), skin (9%), and others (4%). Headache (190, 48.2%), fatigue (71, 18%), loss of appetite (69, 17.5%), burning sensation (52, 13.2%), back pain (50, 12.7%), and itching (44, 11.2%) were the most noticeable adverse effects experienced by the study participants (Table 4).

Of 394 study participants who had adverse effect, majority (150, 38.1%) were on TDF/3TC/EFV ART regimen followed by AZT/3TC/NVP (124, 31.5%) (Table 5).

### 3.5. Coping Strategies

The present study showed that positive emotions, social support seeking, taking other medicines to treat ART adverse effect, information seeking, nonadherence, and holy water were used by 91.1%, 76.6%, 76.6%, 48.7%, 35.5%, and 45.9% of study participants, respectively, as coping strategies for adverse effects of ART (Table 6).

### 3.6. Factors Associated with Coping Strategies Taken by HIV Patients

After controlling different demographic, economical, and other factors through the use of multivariate logistic regression analysis, this study showed that only age, educational status, occupation, and distance were found significantly associated with nonadherence coping strategy for adverse effect of ART (p ≤ 0.05). HIV patients in the age groups of 18–24 years and 25–34 years were more likely to use nonadherence as coping strategy for adverse effect compared to patients' age above 54 years (AOR = 29.54, 95% CI = 2.49–350.25 and AOR = 3.90, 95% CI = 1.24–12.28), respectively.

HIV patients who could not read and write and who had primary school education were more likely to use nonadherence as coping strategy for adverse effect compared to patients who had higher level of education (AOR = 5.70, 95% CI = 2.16-15.05 and AOR = 2.98, 95% CI = 1.19–7.48), respectively.

Respondents who were merchants, farmers, and daily labors and who had no work used nonadherence more likely compared to those who were government employees (AOR = 10.48, 95% CI = 3.82–28.78; AOR = 21.24, 95% CI = 3.74–78.54; AOR = 10.49, 95% CI = 3.30–33.35; and AOR = 4.88, 95% CI = 1.18–20.19), respectively. Respondents who were living far (>20 km) from the hospital used nonadherence coping strategy more likely compared to those who were nearby (≤20 km) (AOR = 2.68, 95% CI = 1.29–5.57) (Table 7).

## 4. Discussion

The present study was aimed at assessing the major adverse effects of ART and coping strategies taken by HIV patients for these adverse effects. About 394 HIV patients who had adverse effects were studied for coping strategy(s) of the adverse effect they faced. They used five coping strategies (positive emotion, social support seeking, information seeking, taking other medications, and nonadherence).

This study revealed that adverse effects were the most common reason for ART regimen changes in HIV patients. Nearly one-third of HIV patients ART regimens were changed due to different reasons based on patient's chart review. Out of 118 regimen changes 73 (61.9%) were due to adverse effects followed by unknown reasons (17) (14.4%), due to treatment failure (16) (13.6%), development of tuberculosis (TB) (11) (9.3%), and pregnancy (1) (0.8%). This regimen change due to adverse effect is higher compared to a study carried out in Jimma University Specialized Hospital (30%). Similarly, treatment failure contributed more to ART regimen change in this study than the above study (0.38%). However, tuberculosis drug interactions and pregnancy were lesser reasons for ART regimen change in this study than Jimma study (17.1%, 3.3%), respectively [24]. The probable reason might be due to variations in ART regimens. Regarding the type of ART regimen, about 91.6% of ART regimens were first line, which is less compared to another study in Debre Markos (98%) [25]. The probable reason for this variation might be because patients may get different strength of counseling regarding the importance of medication adherence.

The present study revealed that a total of 880 ART adverse effects were reported in 394 HIV patients. On average each patient had a chance of facing three adverse effects from their ART regimen which is in contrast with a study done in Uganda, which reported an average of five side effects [26]. This could probably be due to differences in patient's report of adverse effects. The most common adverse effects based on organ system classification reported by the study participants in this study were CNS and PNS (62%), metabolic disorder (16.4%), gastrointestinal (8.6%), skin (9%), and others (4%). A similar study by Lorio et al. also reported that central nervous system (45%), gastrointestinal (27%), and dermatologic (18%) were the major adverse effects in HIV patients [27]. This might be due to ART regimen. In this study the most common adverse effects reported by the participants were headache (48.2%), fatigability (18%), loss of appetite (17.5%), burning sensation (13.2%), back pain (12.7%), itching (11.2%), and insomnia (10.7%). Some of these adverse effects are similar to other studies done in different countries [26, 28]. In this study fatigability (18%) was almost comparable to a study in San Francisco (17%), but much lower than studies in Uganda (67.5%) and France (73.4%) [7, 26, 28]. Loss of appetite (17.5%) was among the major adverse effects reported by study participants in this study which is almost similar to France study (16.9%), but lower than Ugandan study (53%) [26, 28]. Most HIV patients in this study reported headache (48.2%). This finding is in agreement with other studies by Agu et al. reported that headache (14.6%) in 2012, (9.4%)) in 2013 and France (34.6%) [18, 28, 29]. In this study gastritis (9.6%) was reported by HIV patients. Similarly, a study in India reported that gastritis (13.13%) was among the major adverse effects [30]. A study in Ethiopia, HFSH, reported that the major adverse effect was lipodystrophy (49.2%) (18), but in this study only 3 (0.8%) study participants were reported. The probable reason for the variations might be the type of ART regimen that patients were taking. In HFSH study, majority (80.3%) of patients were taking a regimen that contains Stavudine (D4T) as a first line, but in this study currently none of HIV patients were taking ART regimen containing D4T because of adverse effect (toxicity) [21].

Five main coping strategies for adverse effects of ART were used by HIV patients (positive emotion, social support seeking, nonadherence, information seeking, and taking other medications). The majority (91.1%) of participants in this study used positive emotion coping followed by social support seeking (76.6%), taking other medicines (76.6%), information seeking (48.7%), and nonadherence (35.5%). Contrastingly, a study conducted in San Francisco revealed that nonadherence was the most used coping strategy for adverse effect of ART [7]. The reason for the variations might be due to the current functional status of study participants. In this study, almost all (98.7%) study participants were working and only 1.3% had ambulatory functional status compared to a study in San Francisco, where 71% of study participants were not functionally working.

Although majority (91.1%) of the study participants were using positive emotion, there were also a significant number of patients (35.5%) who used nonadherence as a coping strategy in this study. Similarly, in other studies, 17% (France) [28], 14.4% (Nigeria) [18], and 27% (Uganda) [26] of HIV patients used nonadherence as coping strategy for adverse effect of ART.

In this study information seeking (48.7%) was less likely used by HIV patients compared to Uganda study (91.2%). This variation might be due to educational status variations. In this study (21.3%) HIV patients could not read and write compared to only 4.8% who never had formal education.

In the present study participant's age was significantly associated with nonadherence as a coping strategy. Study participants aged between 18 and 24 years and 25 and 34 years were more likely to use nonadherence as coping strategy for adverse effect than those who were above 54 years which is similar to a study in Nigeria [29]. It is inconsistent with another study finding in Uganda, which reported that nonadherence to ART was not statistically significant with any demographic factors [26]. The probable reasons might be due to the fact that young people did not worry about the consequences of being nonadherent to their ART regimen.

Study participants who were merchants, farmers, and daily laborers and those who had no work used nonadherence as a coping strategy more likely than those who were government employees [29]. This is inconsistent with study in Arsi zone, Oromia, where employment was associated with nonadherence [23]. The possible reasons might be indirectly related to educational status, being busy at work, and being worried about having no work. This finding is also supported by different studies [23, 31]. Study participants who could not read and write and who were learning in primary schools were more likely to use nonadherence as coping strategy for adverse effect than those who were above 12^th^ grade. This is in agreement with study in Kenya, which stated that educational status was associated with nonadherence as a coping strategy [31].

Distance from study participant's home to the health institution (UoGRH) was also significantly associated with nonadherence coping strategy. Those who accessed the health institution far away (>20 km) from their home were found to more likely use nonadherence as a coping strategy than those who were nearby (≤20 km). This is in line with a study done in Nigeria, which found that those who accessed the health institution far away from their home were found to be nonadherent [32]. This is inconsistent with study done in Arsi zone [23]. These variations might be because distance was categorized as those who were in the town Gondar (20 km) and outside Gondar town (>20 km) transport problem and cost for transport, but in Arsi those in walking (5 km) and beyond 5 km.

### 4.1. Limitations of the Study

The present study does have some limitations. Due to financial constraints coping strategies were assessed through a self-reporting questionnaire only, which may overestimate the response. The second major limitation of this study is that the respondents consisted only of patients who actually went to ART clinics. We did not interview people who were picking up ART drugs for someone else and this means that we may have missed patients too sick to attend appointments. The final major limitation of this study was the wider confidence interval in the first age category. This resulted from relatively smaller sample size in this age group.

## 5. Conclusion

The present study revealed that positive emotion coping was the most commonly used strategy. Age, level of education, and distance from health institution were the predictors of nonadherence coping strategy.

## Figures and Tables

**Table 1:** Sociodemographic characteristics of the study participants.

Characteristics	Category	Frequency (%)
Age	18-24 years	11 (2.8)
	25-34 years	130 (33)
	35-44 years	150 (38.1)
	45- 54 years	78 (19.8)
	>54 years	25 (6.3)

Educational status	Can't read & write	84 (21.3)
	Can read & write	53 (13.4)
	Grade 1-8^th^	100 (25.4)
	Grade 9-12^th^	81 (20.6)
	College/university	76 (19.3)

Occupation	Government employee	87 (22.1)
	Merchant	99 (25.1))
	Farmer	30 (7.6)
	Student	7 (1.8)
	Daily laborer	65 (16.5)
	Housewife	84 (21.3)
	Others^*∗*^	22 (5.6)

Marital status	Single	66 (16.8)
	Married	204 (51.8)
	Divorced	65 (16.5)
	Widowed	59 (15)

Ethnicity	Amhara	309 (78.4)
	Kimant	58 (14.7)
	Tigrie	24 (6.1)
	Awi	3 (0.8)

^*∗*^Retired and those who had no work.

**Table 2:** Clinical characteristics and health conditions among patients with HIV/AIDS.

**Characteristics**	**Category**	**Frequency (**%**)**
WHO clinical stage	Stage I	93 (23.6)
	Stage II	75 (19)
	Stage III	173 (43.9)
	Stage IV	53 (13.5)
Treatment (T) stage	T1	377 (95.7)
	T2	12 (3)
	T3	5 (1.3)
Baseline functional status	Working	307 (77.9)
	Ambulatory	61 (15.5)
	Bedridden	26 (6.6)
Current functional status	Working	389 (98.7)
	Ambulatory	5 (1.3)
	Bedridden	0 (0)

**Table 3:** Documented reasons for changing ART regimens at University of Gondar.

**Reasons of ART changes**	1^**st**^** change (**%**)**	2^**nd**^** change (**%**)**	3^**rd**^** change (**%**)**
(1) Adverse effect	73 (61.9)	10 (45.5)	3 (30)
(2) TB	11 (9.3)	0 (0)	0 (0)
(3) Pregnancy	1 (0.8)	1 (4.5)	0 (0)
(4) Rx. failure	16 (13.6)	5 (22.7)	0 (0)
(5) Unknown	17 (14.4)	1 (4.5)	1 (10)
(6) Unavailability	0 (0)	5 (22.7)	6 (60)

**N**	**118**	**22**	**10**

**Table 4:** Frequency distribution of adverse effects experienced by HIV patients who wereattending HIV clinic at UoGRH.

	**Type of Adverse effects**	**Number of HIV patients**	**Number of adverse effects**
		**N (**%**)**	**N (**%**)**
Central Nervous	Headache	190 (48.2)	190 (21.6)
System (CNS) &	Sedation	12 (3)	12 (1.4)
Peripheral nervous	Hallucination	25 (6.3)	25 (2.8)
system (PNS)	Anxiety	42 (10.7)	42 (4.8)
	Nervousness	26 (6.6)	26 (3)
	Insomnia	42 (10.7)	42 (4.8)
	Forgetfulness	26 (6.6)	26 (3)
	Vertigo	36 (9.1)	36 (4.1)
	Tinnitus	14 (3.6)	14 (1.6)
	Back pain	50 (12.7)	50 (5.7)
	Peripheral numbness	30 (7.6)	30 (3.4)
	Burning sensation	52 (13.2)	52 (5.9)
**Total CNS & peripheral adverse effects**			**545 (62)**

Gastrointestinal (GI)	Gastritis	38 (9.6)	38 (4.3)
	Vomiting	12 (3)	12 (1.4)
	Diarrhea	10 (2.5)	10 (1.1)
	Nausea	16 (4.1)	16 (1.8)
**Total GI adverse effects**			**76 (8.6)**

Skin	Skin rash	21 (5.3)	21 (2.4)
	Itching	44 (11.2)	44 (5)
	Sweating	14 (3.6)	14 (1.6)
**Total skin adverse effects**			**79 (9)**

Metabolic	Loss of appetite	69 (17.5)	69 (7.8)
	Fatigue	71 (18)	71 (8.1)
	Lipodystrophy	3 (0.8)	3 (0.3)
	Hyperglycemia	2 (0.5)	2 (0.2)
**Total Metabolic adverse effects**			**145 (16.4)**

Others	Anemia	8 (2)	8 (0.9)
	Vision problem	13(3.3)	13(1.5)
	Loss of sexual desire	3 (0.8)	3 (0.3)
	Bloating	5 (1.3)	5 (0.6)
	Crampy abdominal pain	6 (1.5)	6 (0.7)
**Total other adverse effects**			**35(4)**
**Grand total adverse effects**			**880 (100)**

**Table 5:** Cross-tabulation between current ART regimens and adverse effects reported byHIV patients who were attending UoGRH.

		**Current ART Regimens**								
**Adverse effects**	N (%)	AZT/3TC/EFV	AZT/3TC/NVP	TDF/3TC/EFV	TDF/3TC/NVP	ABC/3TC/EFV	TDF/3TC/ATV/r	ABC/3TC/ATV/r	AZT/3TC/ATV/r	AZT/3TC/ABC
**Total pts.**	**394 (100)**	**43 (10.8)**	**124 (31.5)**	**150 (38.1)**	**41 (10.4)**	**1 (0.3)**	**10 (2.5)**	**14 (3.6)**	**9 (2.3)**	**2 (0.5)**

Headache										
Yes	190 (48.2)	30 (69.8)	51 (41.1)	73 (48.7)	20 (48.8)	1 (100)	4(40)	6 (42.9)	4 (44.4)	1 (50)
No	204 (51.8)	13 (30.2)	73 (58.9)	77 (51.3)	21 (51.2)	0 (0)	6 (60)	8 (57.1)	5 (55.6)	1 (50)

Loss of appetite										
Yes	69 (17.5)	8 (18.6)	18 (14.5)	24 (16)	7 (17.1)	0 (0)	3 (30)	6 (42.9)	2 (22.2)	1 (50)
No	325 (82.5)	35 (81.4)	106 (85.5)	126 (84)	34 (82.9)	1 (100)	7 (70)	8 (57.1)	7 (77.8)	1 (50)

Fatigability										
Yes	71 (18)	6 (14)	25 (20.2)	27 (18)	5 (12.2)	0 (0)	3 (30)	4 (28.6)	1 (11.1)	0 (0)
No	323 (82)	37 (86)	99 (79.8)	123 (82)	36 (87.8)	1 (100)	7 (70)	10 (71.7)	8 (88.9)	2 (100)

Burning sensation										
Yes	52 (13.2)	8 (18.6)	19 (15.3)	15 (10)	4 (9.8)	0 (0)	0 (0)	3 (21.4)	2 (22.2)	1 (50)
No	342 (86.8)	35 (81.4)	105 (84.7)	135 (90)	37 (90.8)	1 (100)	10 (100)	11 (78.6)	7 (77.8)	1 (50)

Back pain										
Yes	50 (12.7)	8 (18.6)	14 (11.3)	16 (10.7)	6 (14.6)	0 (0)	3 (30)	1 (7.1)	2 (22.2)	0 (0)
No	344 (87.3)	35 (81.4)	110 (88.7)	134 (89.3)	35 (85.4)	1 (100)	7 (70)	13 (92.9)	7 (77.8)	2 (100)

Insomnia										
Yes	42 (10.7)	5 (11.6)	10 (8.1)	22 (14.7)	3 (7.3)	0 (0)	2 (20)	0 (0)	0 (0)	0 (0)
No	352 (89.3)	38 (88.4)	114 (91.9)	128 (85.3)	38 (92.7)	1 (100)	8 (80)	14 (100)	9 (100)	2 (100)

Anxiety										
Yes	42 (10.7)	4 (9.3)	6 (4.8)	26 (17.3)	5 (12.2)	0 (0)	1 (10)	0 (0)	0 (0)	0 (0)
No	352 (89.3)	39 (90.7)	118 (95.2)	124 (82.7)	36 (87.8)	1 (100)	9 (90)	14 (100)	9 (100)	2 (100)

Itching										
Yes	44 (11.2)	2 (4.7)	17 (13.7)	12 (8)	9 (22)	0 (0)	0 (0)	2 (14.3)	2 (22.2)	0 (0)
No	350 (88.8)	41 (95.3)	107 (86.3)	138 (92)	32 (78)	1 (100)	10 (100)	12 (85.7)	7 (77.8)	2 (100)

**Table 6:** Responses for each coping strategy for adverse effects of ART by HIV patientswho were attending HIV clinic at UoGRH.

**Coping strategy**	**Responses**	
	Yes (%)	No (%)
Positive emotion	359 (91.1)	35 (8.9)
Seeking social support	302 (76.6)	92 (23.4)
Non-adherence	140 (35.5)	254 (64.5)
Information seeking	192 (48.7)	202 (51.3)

Take other medicines	302 (76.6)	92 (23.4)
Holy water^*∗∗*^	181 (45.9)	213 (54.1)

^*∗∗*^Used with the five main coping strategies.

**Table 7:** Univariate and multivariate analysis of factors associated with nonadherence coping strategy at UoGRH.

**Variables**	**Non-adherence coping**			**COR (95**%** CI)**	**AOR (95**%** CI)**	**P – value**
	**Yes (**%**)**	**No (**%**)**	**Total (n)**			
**Age (years)**						
18 – 24	10 (90.9)	1 (9.1)	11	31.66 (3.33 – 300.81)	29.54 (2.49 - 350.25)	0.007^*∗*^
25 – 34	71 (54.6)	59 (45.4)	130	3.81 (1.43 – 10.16)	3.90 (1.24 – 12.28)	0.020^*∗*^
35 – 44	42 (28)	108 (72)	150	1.23 (0.46 – 3.30)	1.51 (0.48 - 4.79)	0.485
45 – 54	11 (14.1)	67 (85.9)	78	0.52 (0.17 - 1.59)	0.44 (0.12 - 1.62)	0.219
>54	6 (24)	19 (76)	25	1	1	

**Occupation**						
Government employee	8 (9.2)	79 (90.8)	87	1	1	
Merchant	56 (56.6)	43 (43.4)	99	12.86 (5.61 – 29.45)	10.48 (3.82 - 28.78)	0.001^*∗*^
Farmer	18 (60)	12 (40)	30	14.81 (5.28 – 41.52)	21.24 (5.74 - 78.54)	0.001^*∗*^
Student	2 (28.6)	5 (71.4)	7	3.95 (0.65 – 23.75)	7.18 (0.93 - 55.65)	0.059
Daily labourer	32 (49.2)	33 (50.8)	65	9.57 (3.99 – 22.96)	10.49 (3.30 - 33.35)	0.001^*∗*^
Housewife	17 (20.2)	67 (79.8)	84	2.51 (1.02 – 6.17)	2.69 (0.89 - 8.10)	0.079
Retired & had no work	7 (31.8)	15 (68.2)	22	4.61 (1.45 – 14.63)	4.88 (1.18 - 20.19)	0.029^*∗*^

**Educational status**						
Can't read & write	45 (53.6)	39 (46.4)	84	5.11 (2.48 – 10.51)	5.70 (2.16 - 15.05)	0.001^*∗*^
Can read & write	26 (49.1)	27 (50.9)	53	4.26 (1.93 – 9.41)	2.02 (0.73 - 5.59)	0.175
Primary (1-8^th^)	46 (46)	54 (54)	100	3.77 (1.87 – 7.60)	2.98 (1.19 - 7.48)	0.020^*∗*^
Secondary (9 -12^th^)	9 (11.1)	72 (88.9)	81	0.55 (0.22 – 1.37)	0.38 (0.13 - 1.13)	0.083
Above 12^th^	14 (18.4)	62(81.6)	76	1	1	

**Distance (km)**						
1 – 20	108 (33.2)	217 (66.8)	325	1	1	
>20	32 (46.4)	37 (53.6)	69	1.74 (1.03 – 2.94)	2.68 (1.29 - 5.57)	0.008^*∗*^

COR = crude odds ratio, AOR = adjusted odds ratio, and CI = confidence interval.

## Data Availability

The data used to support the findings of this study are available from the corresponding author upon request.

## Abbreviations and Acronyms

3TC: Lamivudine
ABC: Abacavir
ADR: Adverse drug reaction
AIDS: Acquired Immune Deficiency Syndrome
AOR: Adjusted odds ratios
ART: Antiretroviral therapy
ATV/r: Atazanavir/ritonavir
AZT: Zidovudine
CD4: Cluster of Differentiation 4
CI: Confidence interval
EFV: Efavirenz
HIV: Human immunodeficiency virus
IQR: Interquartile range
NVP: Nevirapine
QoL: Quality of life
SD: Standard deviation
SPSS: Statistical Package for Social Sciences
TDF: Tenofovir Disoproxil Fumarate
UoGRH: University of Gondar Referral Hospital
WHO: World Health Organization.
